# Essential role of the Cdk2 activator RingoA in meiotic telomere tethering to the nuclear envelope

**DOI:** 10.1038/ncomms11084

**Published:** 2016-03-30

**Authors:** Petra Mikolcevic, Michitaka Isoda, Hiroki Shibuya, Ivan del Barco Barrantes, Ana Igea, José A. Suja, Sue Shackleton, Yoshinori Watanabe, Angel R. Nebreda

**Affiliations:** 1 Institute for Research in Biomedicine (IRB Barcelona), Barcelona Institute of Science and Technology, Barcelona 08028, Spain; 2 Laboratory of Chromosome Dynamics, Institute of Molecular and Cellular Biosciences, University of Tokyo, Yayoi, Tokyo 113-0032, Japan; 3 Unidad de Biología Celular, Departamento de Biología, Facultad de Ciencias, Universidad Autónoma de Madrid, Madrid 28049, Spain; 4 Department of Molecular and Cell Biology, Henry Wellcome Building, University of Leicester, Leicester LE1 9HN, UK; 5 Institució Catalana de Recerca i Estudis Avançats (ICREA), Barcelona 08010, Spain

## Abstract

Cyclin-dependent kinases (CDKs) play key roles in cell cycle regulation. Genetic analysis in mice has revealed an essential role for Cdk2 in meiosis, which renders Cdk2 knockout (KO) mice sterile. Here we show that mice deficient in RingoA, an atypical activator of Cdk1 and Cdk2 that has no amino acid sequence homology to cyclins, are sterile and display meiotic defects virtually identical to those observed in Cdk2 KO mice including non-homologous chromosome pairing, unrepaired double-strand breaks, undetectable sex-body and pachytene arrest. Interestingly, RingoA is required for Cdk2 targeting to telomeres and RingoA KO spermatocytes display severely affected telomere tethering as well as impaired distribution of Sun1, a protein essential for the attachment of telomeres to the nuclear envelope. Our results identify RingoA as an important activator of Cdk2 at meiotic telomeres, and provide genetic evidence for a physiological function of mammalian Cdk2 that is not dependent on cyclins.

## Abstract (web summary)

CDKs play central roles in cell cycle regulation and are normally activated by cyclins. Here the authors show that RingoA induces a cyclin-independent function of CDK2 at meiotic telomeres, which regulates their tethering to the nuclear envelope and proper synapsis of homologous chromosomes.

## Graphical abstract

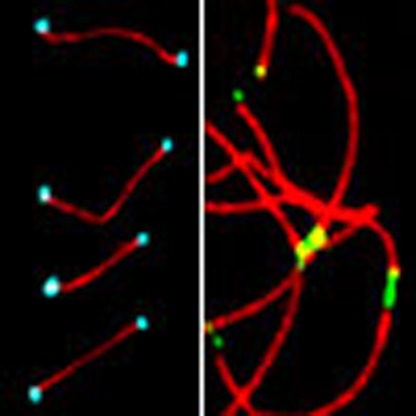

Cell division is orchestrated by the periodical activation of cyclin-dependent kinases (CDKs) whose activity is modulated by the binding of regulatory subunits named cyclins^1^. However, there is evidence that CDK activation does not always require the binding of conventional cyclins^2^. Atypical CDK activators include the RINGO/Speedy proteins, which were initially identified as potent inducers of meiotic maturation in *Xenopus* oocytes^3^,^4^. *Xenopus* RINGO (XRINGO) can interact with and directly activate both Cdk1 and Cdk2, despite having no homology in its amino acid sequence to cyclins^3^. The activation of Cdk1 and Cdk2 by XRINGO is independent of the control mechanisms that regulate CDK/cyclin complexes^5^. Interestingly, XRINGO-activated Cdk1 and Cdk2 have altered substrate specificity and can phosphorylate the CDK inhibitory kinase Myt1 on three specific Ser residues much more efficiently than the cyclin-activated CDKs^6^. These phosphorylations inhibit the catalytic activity of Myt1, which probably accounts for the role of XRINGO in *Xenopus* oocyte maturation^6^,^7^,^8^. Thus, RINGO proteins are novel non-cyclin CDK regulators that may allow CDKs to play different roles when cyclin expression is compromised or to bypass control mechanisms of CDK-cyclin complexes.

RINGO proteins are conserved among metazoans and several mammalian RINGO family members have been identified, which can all associate with and regulate Cdk1 and Cdk2 (refs 9, 10). The best-studied family member in mammals is RingoA (also known as SpdyA, Spy1 or Ringo3). Overexpression of RingoA in mammalian cell lines enhances the rate of cell proliferation^11^, but high levels of RingoA can interfere with cytokinesis and chromosome decondensation, probably because of its ability to maintain high Cdk1 activity in mitosis^12^. Experiments using cell lines and ectopically expressed proteins have implicated RingoA in processes, such as checkpoint signalling and tumorigenesis^13^,^14^,^15^, but the precise physiological functions of RingoA remain elusive.

Generation of knockout (KO) mice for CDKs and cyclins during the past decade has shown that only Cdk1 is essential for mouse cell proliferation as it can compensate for other CDKs by binding to several types of cyclins in a cell cycle stage-dependent manner^16^. In contrast, Cdk2 is dispensable for somatic cell proliferation in mice but essential for male and female meiosis^17^,^18^. Interestingly, the meiotic phenotype of Cdk2 KO mice is different from the reported phenotypes for cyclin-deficient mice, which show later (cyclins A1 and A2) or restricted to males (cyclins E1 and E2) meiotic defects^19^,^20^,^21^. In the case of B-type cyclins, mice deficient in cyclin B1 die early in embryogenesis, while cyclin B2 has no role in meiosis and cyclin B3 appears to inhibit prophase I^22^,^23^. It therefore seems that meiotic Cdk2 might be regulated by a cyclin-independent mechanism, which prompted us to investigate the role of RingoA in meiosis. We have found that RingoA KO mice phenocopy the meiotic defects of Cdk2 KO mice, indicating that RingoA is an essential activator of Cdk2 in meiosis. We also provide evidence that Cdk2-RingoA regulates the inner nuclear membrane protein Sun1 for meiotic telomere tethering to the nuclear envelope (NE), a prerequisite for chromosome pairing and successful completion of the first meiotic prophase.

## Results

### RingoA KO mice are sterile

**Figure 1:**
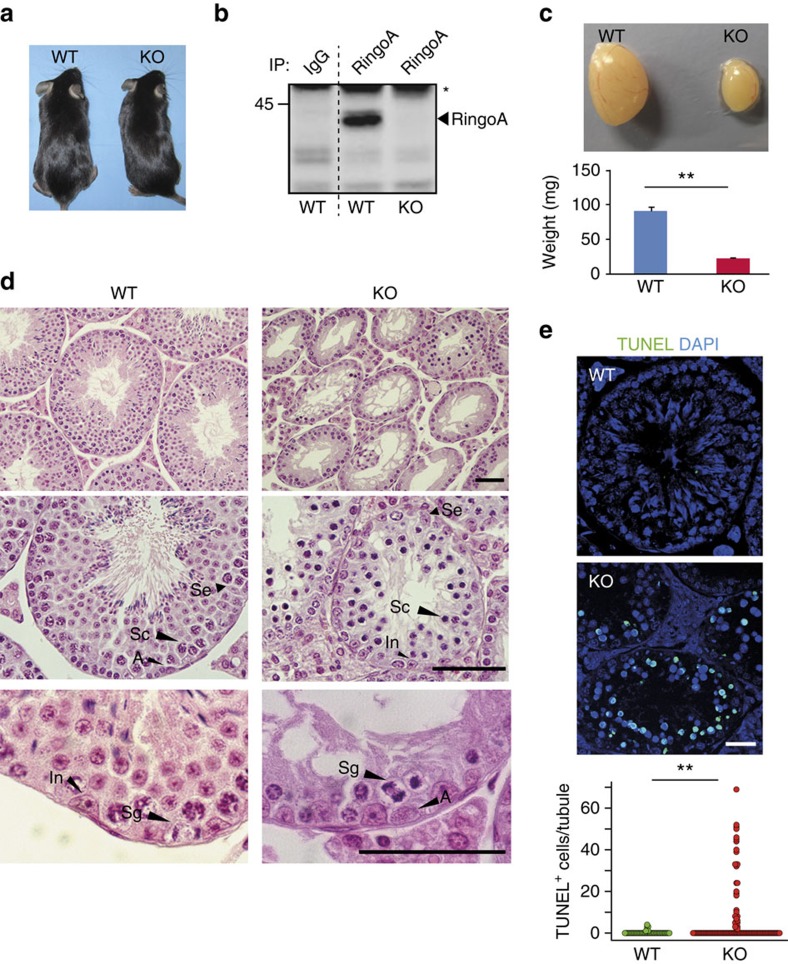
RingoA KO males are sterile. (**a**) RingoA-deficient mice are indistinguishable from their littermates. (**b**) RingoA immunoprecipitates from WT and KO testis lysates were immunoblotted for RingoA. The asterisk indicates IgGs. (**c**) RingoA KO testes are about four times smaller than the WT testes in 2-month-old mice. (*n*=9 WT and 5 KO, *P* value=0.001). (**d**) Testis from 2-month WT and KO mice were fixed in Bouin's solution, embedded in paraffin and sections (6 μm) were stained with H&E. A, A-type spermatogonia; In, intermediate spermatogonia; Sc, spermatocytes; Se, Sertoli cell; Sg, spermatogonia in anaphase. Scale bar, 50 μm. (**e**) Paraffin sections from WT and KO testes were stained with TUNEL (green) and DAPI (blue). Scale bar, 10 μm. TUNEL^+^ spermatocytes per seminiferous tubule were counted. (*n*=48 WT and 114 KO tubules, *P* value=0.009). Error bars are presented as the average with s.d. Asterisks indicate statistical significance (**P value<0.01) determined by the unpaired two-way Wilcoxon rank-sum test. The uncropped immunoblot is shown in Supplementary Fig. 7.

To genetically inactivate RingoA, we generated mice carrying a floxed version of the *Spyda* gene, which encodes RingoA (Supplementary Fig. 1). Mice with the *Spdya*^lox^ allele were crossed with Sox2-Cre transgenic mice to delete exon 3 and generate *Spdya*^*+/−*^ mice. The heterozygous mice were healthy and fertile and were inter-crossed to produce RingoA KO mice. These mice were born with the expected Mendelian frequency (Supplementary Table 1), indicating that RingoA is not essential for embryonic development, and we did not detect any differences with adult wild-type (WT) littermates (Fig. 1a). However, both RingoA KO males and females were found to be sterile. We confirmed by PCR with reverse transcription and western blotting that RingoA KO testis lacked RingoA expression (Fig. 1b and Supplementary Fig. 2).

The testes from 2-month-old RingoA KO mice were hypoplastic and about four times smaller in size and weight than those from WT littermates (Fig. 1c). Histological examination of RingoA-deficient testes revealed that the epithelium of the majority of the seminiferous tubules was abnormal and contained only a single layer of Sertoli and spermatogonial cells (Fig. 1d, top row). Moreover, the seminiferous tubules were narrower and lacked post-meiotic germ cells (spermatozoa and spermatids) showing also degenerating spermatocyte-like cells with highly condensed nuclei (Fig. 1d, Sc). The condensed nuclei resembled the chromatin degradation characteristic of apoptosis (Fig. 1d, Sc) and KO animals contained an increased number of TUNEL positive cells per seminiferous tubule compared with WT mice (Fig. 1e). The spermatogonial compartment of RingoA KO testes appeared normal (Fig. 1d, Sg) and anaphase B-type spermatogonia were observed close to the basal lamina (Fig. 1d, In and A). Altogether, these data indicate that the spermatogenesis of RingoA KO mice was arrested at stage IV, in mid-pachytene of meiosis I^24^.

Ovaries of the RingoA KO female mice were also atrophic with most of the organ composed of interstitial and stromal cells (Supplementary Fig. 3a). We were unable to detect any ovarian follicles in the RingoA KO mice.

### RingoA KO spermatocytes arrest at a pachytene-like stage

In prophase of meiosis I, homologous chromosomes pair and recombine to form bivalents, which can be monitored by staining for components of the synaptonemal complex (SC). The axial/lateral elements of SC start to form in leptotene and SCs are fully established in pachytene, before they disassemble in diplotene for chromosome condensation.

**Figure 2:**
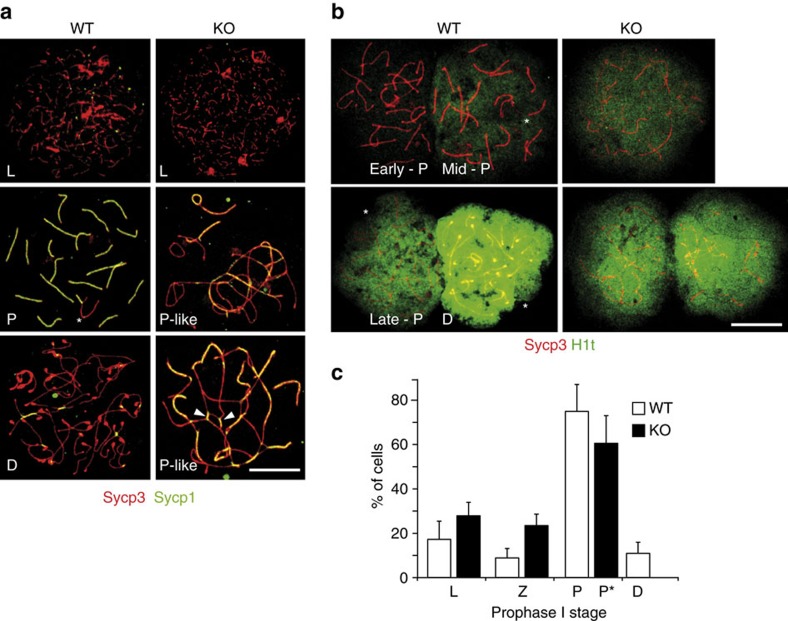
RingoA KO spermatocytes arrest in a pachytene-like stage with aberrant synapsis. (**a**) WT and KO spread spermatocytes were immunolabelled for Sycp3 (red) and Sycp1 (green). KO spermatocytes presented non-homologous pairing and partner switching (arrowheads). (**b**) WT and KO spread spermatocytes were immunolabelled for Sycp3 (red) and histone H1t (green). On the basis of H1t signal intensity, some pachytene-like KO spermatocytes reached mid-pachytene (93%) and late-pachytene (7%) stages. (**c**) Percentage of cells at each stage of prophase I in the WT and KO spreads of 2-months-old testes (based on stainings as in **a**; *n*=3, 150 spermatocytes per mouse). D, diplotene; L, leptotene; P, pachytene; P*, pachytene-like; Z, zygotene. The sex-body is indicated in the WT pachytene spermatocytes (star). Scale bar, 10 μm.

In nuclear spreads from WT testes, the homologous chromosomes were fully synapsed and the SC was established by pachytene, as seen by a fully overlapping signal of the axial/lateral element protein Sycp3 and the transverse filament protein Sycp1 (Fig. 2a). The chromosomes of the most advanced prophase I spermatocytes found in RingoA KO seminiferous tubules showed elongated stretches of Sycp3 co-localizing with Sycp1, which indicates synapsed regions (Fig. 2a). However, the synapsed regions in these spermatocytes showed frequent partner switching, indicating extensive non-homologous pairing (Fig. 2a, arrowheads). The same phenotype was observed for RingoA KO oocytes (Supplementary Fig. 3b, arrowheads).

Since proper homologous pairing was seriously impaired in RingoA KO spermatocytes, it was difficult to conclude in which stage the more advanced prophase I cells were arrested. To address this, we analysed the distribution of the testis-specific histone H1t, a marker of entry into mid-pachytene that gradually increases until diplotene^25^. In WT spermatocytes, we found that the H1t signal marked mid-pachytene chromatin, then increased gradually through late pachytene and was saturated in diplotene (Fig. 2b). The intensity of H1t staining indicated that the majority of the RingoA KO spermatocytes were arrested in early to mid-pachytene (93%) and only some spermatocytes were able to reach late pachytene (7%) (Fig. 2b).

These findings suggest that RingoA KO spermatocytes arrest in a pachytene-like stage, probably due to the activation of the pachytene checkpoint^26^. Quantitative analysis confirmed that the frequency of early prophase I stages was similar in WT and RingoA KO prophase I spermatocytes, but diplotene spermatocytes were absent from the KO, indicating a complete pachytene arrest (Fig. 2c). We did not observe the accumulation of RingoA KO cells in pachytene stage, probably due to the checkpoint-arrested spermatocytes undergoing tubule-wide synchronized apoptosis after prolonged pachytene arrest (see Fig. 1e above), as seen in Cdk2 KO spermatocytes^17^.

### Increased non-repaired DNA in RingoA KO spermatocytes

**Figure 3:**
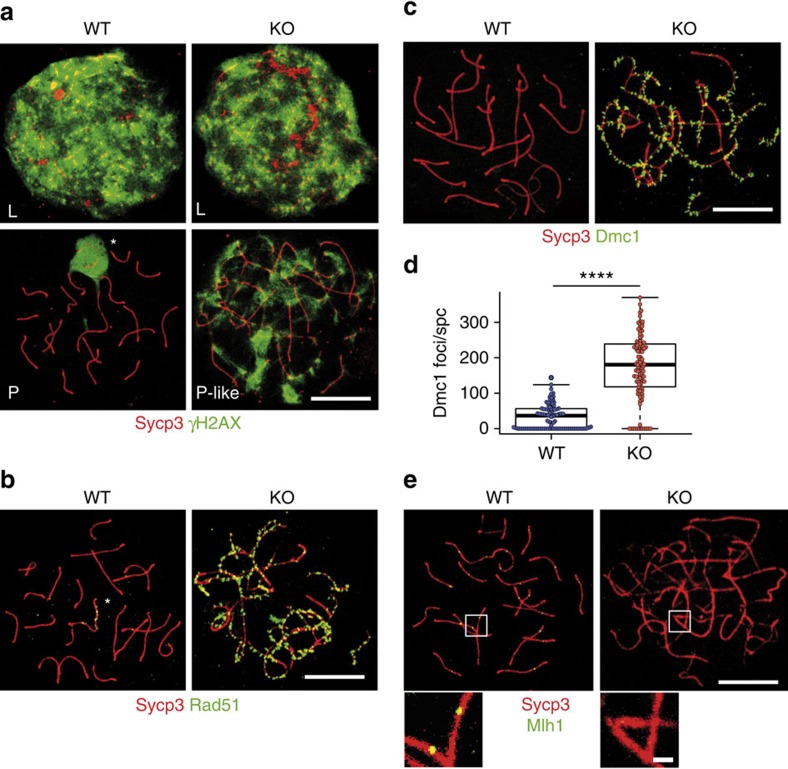
RingoA KO spermatocytes show non-repaired DSBs and lack of crossing-overs. (**a**) WT and KO spread spermatocytes were immunolabelled for Sycp3 (red) and γH2AX (green). γH2AX marked DSBs in leptotene WT and KO spermatocytes. In pachytene WT spermatocytes, DSBs were mostly repaired and γH2AX was sequestered to the sex-body (star), whereas the γH2AX signal persisted in pachytene-like KO spermatocytes. (**b**,**c**) WT and KO spread spermatocytes were immunolabelled for Sycp3 (red) and either Rad51 (green in **b**) or Dmc1 (green in **c**). Rad51 and Dmc1 mark intermediate stages of DSB repair and, in WT pachytene spermatocytes, Rad51 appears as spots along asynapsed regions of the sex chromosomes (**b**, star), whereas Dmc1 gets removed from the chromosomes (**c**). In pachytene-like KO spermatocytes, Rad51 and Dmc1 marked numerous foci along synapsed and asynapsed chromosome regions. (**d**) Quantification of Dmc1 foci in WT (*n*=125 cells, 3 mice) and KO spermatocytes (spc) (*n*=102 cells, 3 mice). *P* value= 2.2e−16. (**e**) WT and KO spread spermatocytes were immunolabelled for Sycp3 (red) and Mlh1 (green). Mlh1 marks late-recombination nodules in WT spermatocytes, whereas no Mlh1 staining was seen in KO spermatocytes (*n*=150, 3 mice). Scale bars, 10 μm; inset, 1 μm. The median is indicated by the thick line and the first and third quartiles of the box plot by thin lines. Asterisks indicate the statistical significance (****P value <0.0001) determined by the unpaired two-way Wilcoxon rank-sum test.

The process of chromosome pairing is facilitated by the introduction of DNA double-strand breaks (DSBs)^27^, which can be monitored by the accumulation of phospho-histone H2AX (γH2AX) positive foci from leptotene until the DSBs are repaired in pachytene. As expected, in WT spermatocytes, the γH2AX signal was high at leptotene and restricted to the sex-body in pachytene spermatocytes (Fig. 3a, left column). However, in RingoA KO spermatocytes, the γH2AX signal was high throughout prophase I but without the typical enrichment at the sex-body (Fig. 3a, right column). This indicates that DSBs were properly introduced, but DSB repair was impaired and sex-body formation was not detected.

After their formation by the topoisomerase-like enzyme Spo11, DSBs are processed for repair and recruit recombination proteins, including Rad51 and Dmc1 (ref. 27). Rad51 and Dmc1 mark DSBs along the axial/lateral elements and upon proper SC formation in pachytene are mostly replaced by other intermediate-stage proteins. In WT pachytene spermatocytes, Rad51 foci remained only along the asynapsed axial elements of the sex chromosomes (Fig. 3b), whereas in RingoA KO spermatocytes Rad51 was widely distributed along the axial/lateral elements of SCs even in pachytene-like stages (Fig. 3b). Similar results were observed for Dmc1 foci (Fig. 3c). We quantified the amount of Dmc1 foci in RingoA KO pachytene-like spermatocytes. We scored on average 35 Dmc1 foci per WT mid-pachytene spermatocyte and 168 Dmc1 foci per RingoA KO pachytene-like spermatocyte (Fig. 3d).

The late-recombination marker MutL homologue 1 (Mlh1) normally appears at the designated crossing-over sites from mid-pachytene onwards^28^. However, we could not detect any Mlh1 foci in RingoA KO pachytene-like spermatocytes (Fig. 3e). We also investigated the Mlh1 staining in oocytes, which have a more permissive pachytene checkpoint. We analysed 130 RingoA KO oocytes, which seemed in later stages of pachytene with longer stretches of thick Sycp3 fibres, and found that ∼5% of those oocytes (7/130) showed an average of five Mlh1 foci of which 84% also co-stained for Cdk2 (Supplementary Fig. 3c). Our results indicate that DSB initiation and the recruitment of early repair proteins is functional in the absence of RingoA, whereas the late-recombination stages are impaired and crossing-over sites are not produced, perhaps as a consequence of improper homologous pairing due to defects upstream of the DSB repair.

### RingoA co-localizes with Cdk2 at telomeres

**Figure 4:**
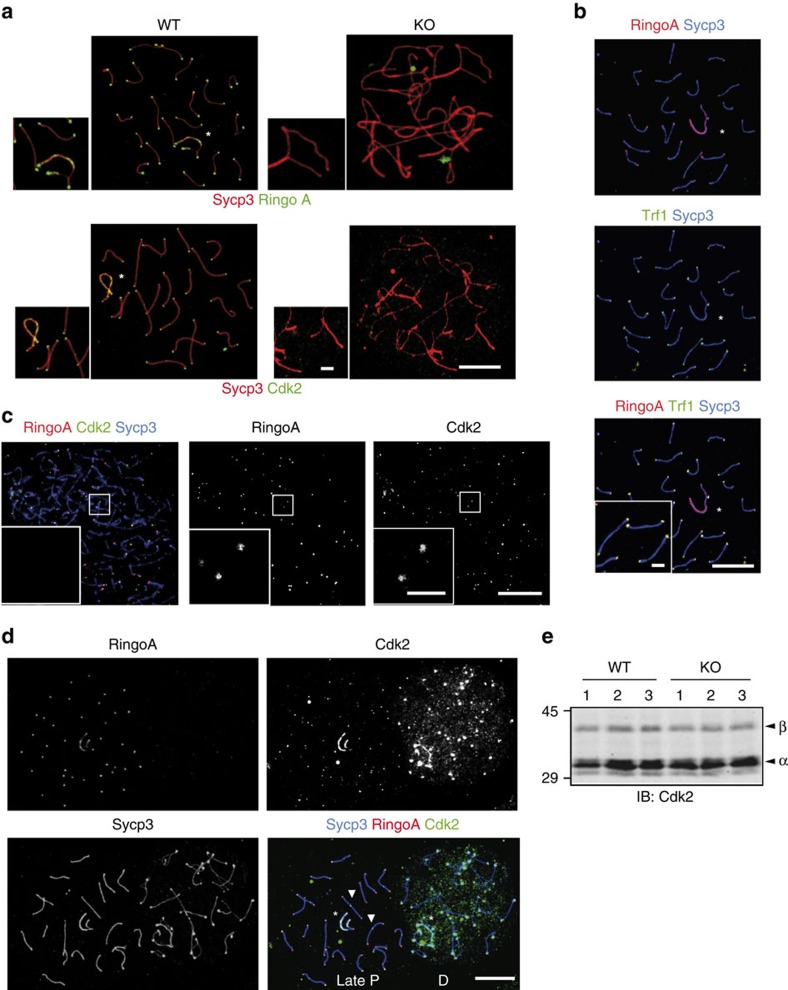
RingoA is required for Cdk2 localization and co-localizes with Cdk2 at telomeres in prophase I spermatocytes. (**a**) WT and KO spread spermatocytes were immunolabelled for Sycp3 (red) and either RingoA (green) or Cdk2 (green). In WT pachytene spermatocytes, RingoA localizes at telomeres and the axial elements of sex chromosomes (star). Cdk2 localization at telomeres, the axial elements of sex chromosomes (star), and at late-recombination nodules is completely lost in KO spermatocytes. RingoA and Cdk2 are not detected in KO pachytene-like spermatocytes. (**b**) WT pachytene spread spermatocytes were immunolabelled for Sycp3 (blue), RingoA (red) and Trf1 (green). RingoA co-localizes with Trf1 at telomeres. (**c**) WT leptotene spread spermatocytes were immunolabelled for Sycp3 (blue), RingoA (red) and Cdk2 (green). Cdk2 and RingoA co-localize at the telomeric regions. (**d**) WT late pachytene and diplotene spermatocyte spreads were immunolabelled for Sycp3 (blue), RingoA (red) and Cdk2 (green). RingoA and Cdk2 co-localize until late pachytene. RingoA is absent upon entry into diplotene, while Cdk2 signal gets more dispersed within the nucleus. (**e**) Testis lysates from 18 dpp WT (*n*=3) or RingoA KO (*n*=3) mice were analysed by immunoblotting with Cdk2 antibody. The arrowheads indicate Cdk2β (upper) and Cdk2α (lower). Scale bars, 10 μm; inset, 2 μm. The uncropped immunoblot is shown in Supplementary Fig. 7.

The defective homologue pairing phenotype observed in RingoA KO spermatocytes is strikingly similar to what has been described in Cdk2 KO spermatocytes, suggesting that RingoA might be a key meiotic regulator of Cdk2. We found that RingoA localized to the telomeric regions in WT pachytene spermatocytes (Fig. 4a,b), as it has been reported for Cdk2 (refs 17, 29), and co-localized with Cdk2 from leptotene (Fig. 4c) disappearing from telomeres as cells entered the diplotene stage (Fig. 4d). RingoA and Cdk2 also co-localized along the asynapsed axial elements of sex chromosomes in 58% of WT pachytene spermatocytes (Fig. 4d and Supplementary Fig. 4), but RingoA was not found at crossing-over sites (Fig. 4d, arrowheads). RingoA and Cdk2 co-localized until late pachytene after which RingoA signal disappeared completely and Cdk2 signal seemed more dispersed in the diplotene nucleus (Fig. 4d). Interestingly, the localization of Cdk2 in telomeres was abolished in the RingoA KO pachytene-like spermatocytes (Fig. 4a). In spite of the disappearance of Cdk2 from telomeres in RingoA-deficient spermatocytes, total Cdk2 expression levels were similar in WT and RingoA KO testes at 18 dpp, a time point when the WT and KO testes are comparable (Fig. 4e).

**Figure 5:**
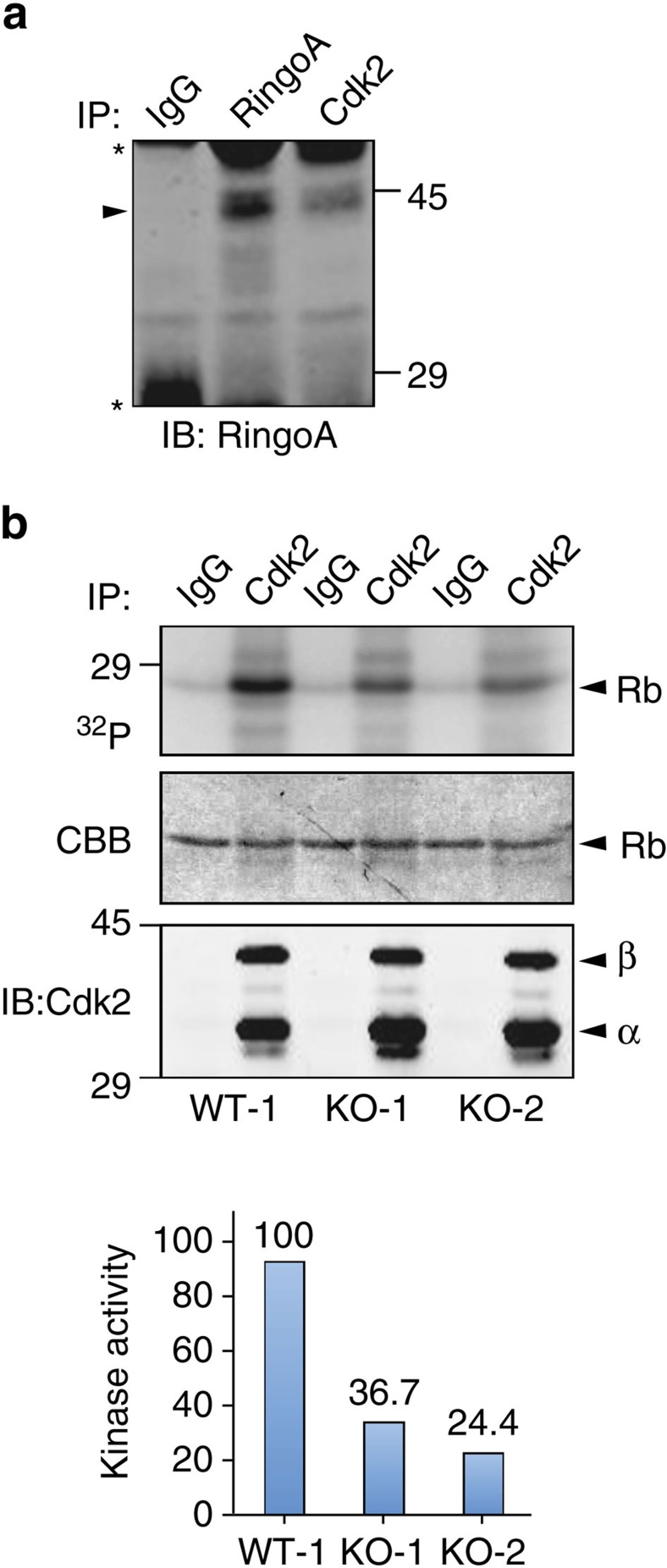
RingoA interacts with Cdk2 and regulates Cdk2 kinase activity in testis. (**a**) Immunoprecipitates from WT testis lysates using RingoA or Cdk2 antibodies and control IgGs were analysed by immunoblotting for RingoA. The arrowhead indicates RingoA and the asterisks IgGs. (**b**) WT and KO 18 dpp old testis lysates were immunoprecipitated with Cdk2 antibodies or control IgGs. The immunoprecipitates were assayed in an *in vitro* kinase assay using γ-^32^P-ATP and Rb protein as a substrate (upper row) and the gel was stained with Coomassie (middle row). An aliquot of the immunoprecipitates was analysed by immunoblotting with Cdk2 antibodies (bottom row). The phosphorylated Rb protein was quantified using ImageJ and was referred to the amount of RB protein detected in the Coomassie staining. The experiments were reproduced two to three times. The uncropped images are shown in Supplementary Fig. 7.

Consistent with the above results, we detected interaction between endogenous RingoA and Cdk2 in testis by co-immunoprecipitation experiments (Fig. 5a). Furthermore, the kinase activity of Cdk2 immunoprecipitated from 18 dpp testes was reduced by ∼70% in the RingoA KO mice compared with WT mice (Fig. 5b), supporting the idea that impaired Cdk2 activity and subcellular localization are likely to account for the defects observed in RingoA KO mice.

### RingoA loss negatively affects meiotic telomere function

Meiotic telomeres are central players of prophase I progression. The initial steps in prophase I are tightly linked to telomeres, although the underlying mechanisms are poorly understood^30^. At the onset of meiosis, telomeres are lengthened by presumably two mechanisms, alternative lengthening of telomeres (ALT) and telomerase^31^,^32^,^33^ and fortified mechanically by the addition of cohesins^34^. This prepares the telomeres for anchoring to the NE, which are then pulled along the NE to form a so-called ‘bouquet' cluster^35^. This is believed to facilitate homologue pairing and govern subsequent steps of prophase I. Hence, interfering with any of these steps will result in aberrant prophase I.

**Figure 6:**
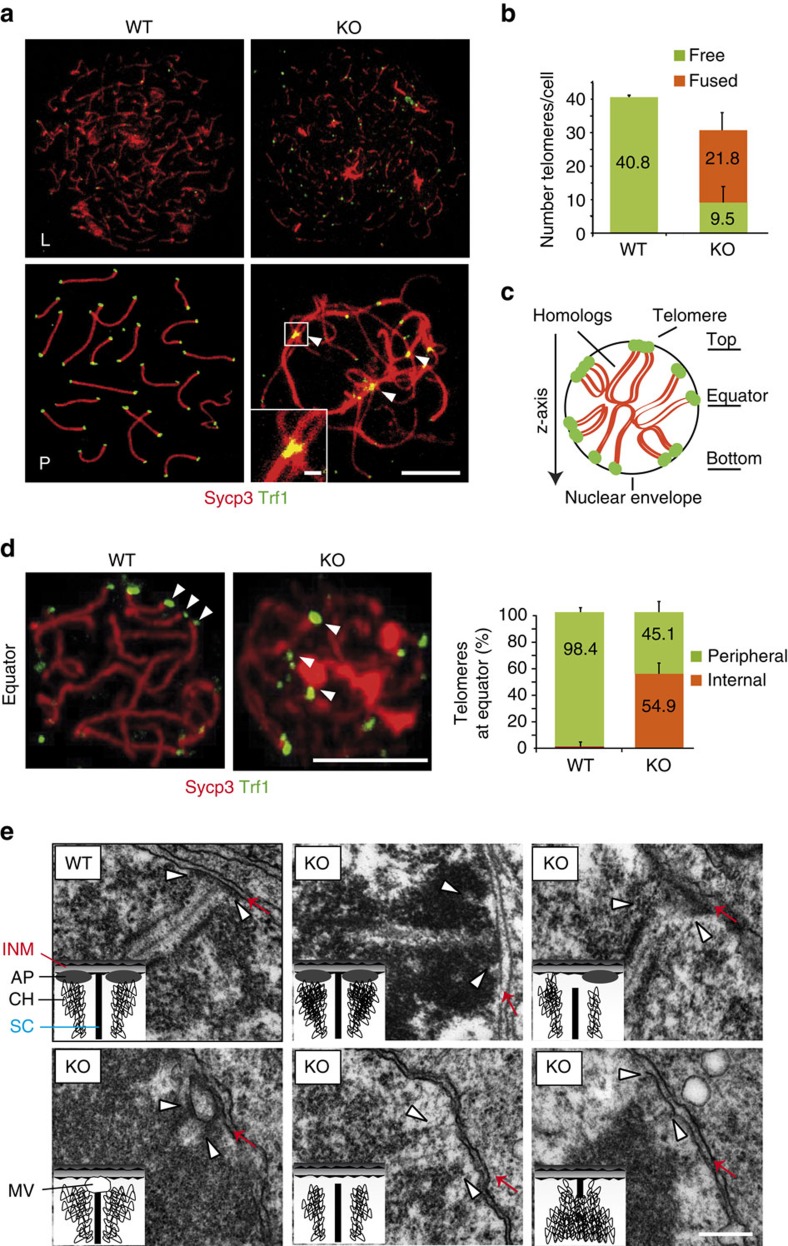
RingoA loss negatively affects meiotic telomere function. (**a**) WT and KO spread spermatocytes were immunolabelled for Sycp3 (red) and Trf1 (green). In pachytene-like KO spermatocytes, Trf1 can be seen along the SC threads, instead of at the ends, indicating chromosome fusions (arrowheads). (**b**) Quantification of the number of free and fused telomeric Trf1 signals in pachytene WT (*n*=27) and pachytene-like KO spermatocytes (*n*=35 cells). Out of 1,100 telomeres scored, two were fused in WT and 763 in KO spermatocytes. Average with s.d. is represented. (**c**) A scheme of a 3D preserved (squashed) pachytene spermatocyte nucleus. (**d**) Squashed WT pachytene and KO pachytene-like spermatocytes were immunolabelled for Sycp3 (red) and Trf1 (green). Telomere Trf1 signals were found at the rim of the cell nucleus at the equator of the WT spermatocyte (tethered to the NE) (arrowheads). In KO spermatocytes, many telomeres (arrowheads) were seen inside the nucleus at the cell equatorial region. Quantification of peripheral and internal telomeres at the equatorial region of squashed WT (*n*=20 cells) and KO (*n*=18 cells) spermatocytes. Average with s.d. is represented. Scale bar, 5 μm. (**e**) Electron micrographs showing telomeres (white arrowheads) and inner nuclear membrane (red arrows) in WT pachytene and KO pachytene-like nuclei, with schematic illustrations of the structures. AP, attachment plate; CH, chromatin; INM, inner nuclear membrane; MV, membrane vesicle; SC, synaptonemal complex. Scale bar, 250 nm.

We found that the DNA telomere repeat binding protein Trf1 localized to the telomeres in RingoA KO spermatocytes (Fig. 6a) and oocytes (Supplementary Fig. 3b). However, about 70% of the telomeres appeared fused together both in spermatocytes (Fig. 6b) and in oocytes (Supplementary Fig. 3b, arrowheads), indicating that the absence of RingoA impaired the integrity of telomeres. To assess the binding of telomeres to the NE, we used squashed spermatocytes to preserve the volume of nuclei and then scored by confocal imaging for Trf1 signals at the circumference of nuclear sections, as an indicator of the telomeres attached to the NE. We obtained z-stacks of entire nuclei and determined the top, equator and bottom planes (Fig. 6c). In WT spermatocytes, telomeres at the cell equator were mostly at the ends of SCs, indicating that they were properly attached to the NE (Fig. 6c,d and Supplementary Movie 1). In contrast, 55% of the telomeres in RingoA KO spermatocytes were scattered within the nucleus, indicating that they were probably not attached to the NE (Fig. 6c,d and Supplementary Movie 2). To test if the remaining 45% of the telomeres that were located close to the NE in RingoA KO spermatocytes were properly attached, we used electron microscopy. We found that around 30% of telomeres found in the vicinity of the NE in RingoA KO spermatocytes were properly attached; the other 70% were unattached and either did not reach the inner nuclear membrane and did not form an attachment plate (AP), or were bound to a membrane vesicle just before reaching the NE (Fig. 6e).

**Figure 7:**
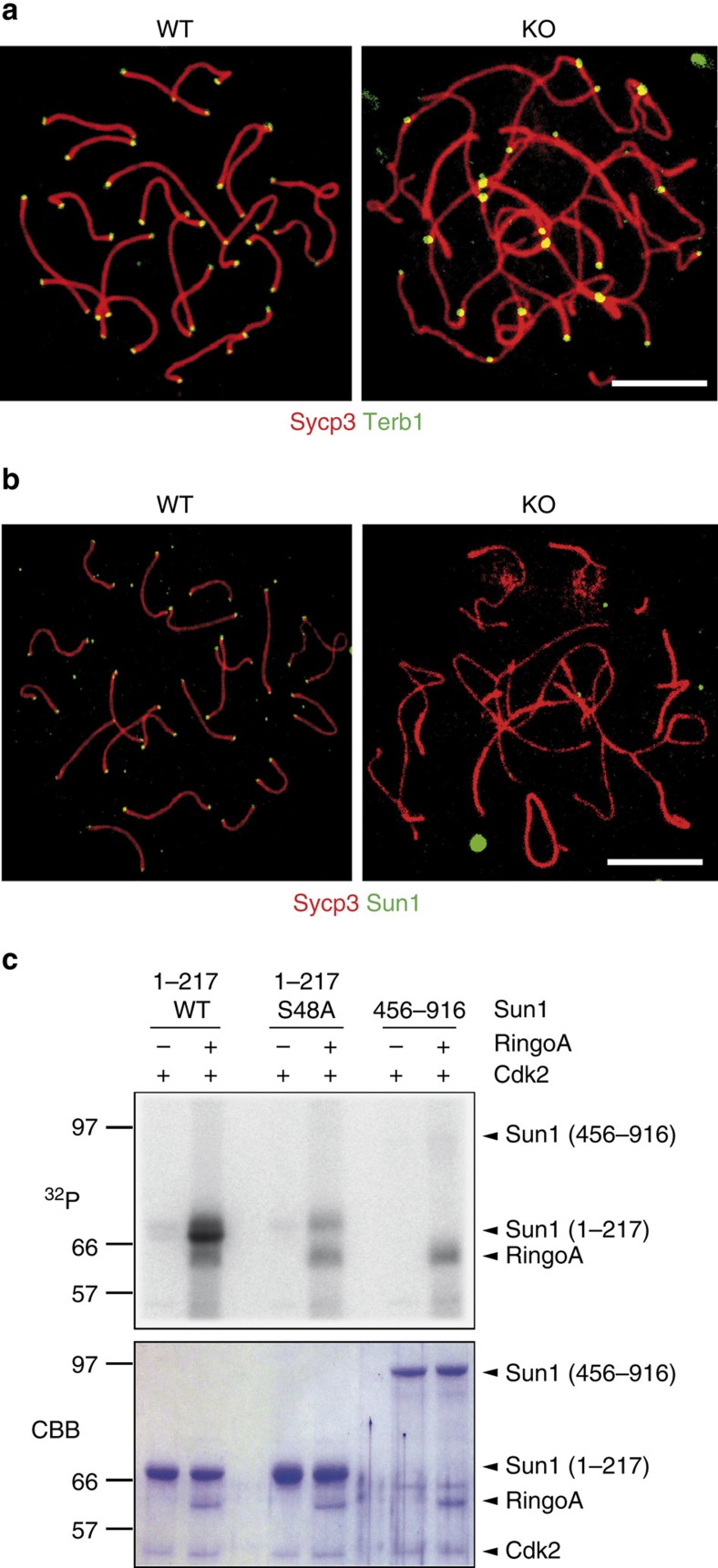
RingoA regulates Sun1 telomeric localization. (**a**) WT and KO spread spermatocytes were immunolabelled for Sycp3 (red) and Terb1 (green). Terb1 signals at telomeres were unperturbed in RingoA KO spermatocytes. (**b**) WT and KO spread spermatocytes were immunolabeled for Sycp3 (red) and Sun1 (green). Telomeric localization of Sun1 was abolished in KO spermatocytes. Scale bar, 10 μm. (**c**) Purified MBP-fused proteins including amino acids 1-217 (either WT or with the mutation S48A) or amino acids 456–916 of Sun1 were incubated with Cdk2 and RingoA in the presence of [γ-^32^P]ATP. Proteins were analysed by SDS–PAGE, stained with Coomassie Brilliant Blue (CBB) and subjected to autoradiography (^32^P). The experiments were reproduced three times. The uncropped autoradiography is shown in Supplementary Fig. 7.

Terb1 and Sun1 are two key proteins linking the meiotic telomeres to the NE. Terb1 participates in telomere fortification by recruiting cohesins to the site^34^, whereas Sun1 tethers telomeres to the inner nuclear membrane^36^. We found that neither the localization of Terb1 (Fig. 7a) nor that of its associated cohesins Smc3 and Rad21L (Supplementary Fig. 5) were affected in the RingoA KO pachytene-like spermatocytes. On the other hand, the absence of RingoA resulted in the lack of detectable telomere-bound Sun1 in spermatocyte spreads (Fig. 7b). However, when analysed in RingoA KO squashed spermatocytes, most Sun1 was detected as a polarized cap, as in Cdk2 KO spermatocytes^37^, with one or two signals sometimes observed within the nuclei (Supplementary Fig. 6). The difference between spermatocyte spreads and squashes indicates that some telomeric Sun1 protein might remain in RingoA KO spermatocytes but not as tightly bound to the telomeres as in the WT spermatocytes. *In vitro* kinase assays using purified recombinant proteins showed that incubation with RingoA substantially boosted the ability of Cdk2 to phosphorylate the N-terminus of Sun1 (Fig. 7c), which is responsible for telomere binding^38^,^39^. Moreover, the phosphorylation of the Sun1 N-terminus by Cdk2-RingoA was strongly reduced by the mutation of Ser48 to Ala (Fig. 7c). It has been reported that Cdk1 can phosphorylate Sun1 on Ser48 in mitosis^40^ but the physiological role of this phosphorylation in meiosis is unknown.

### Disrupted telomere architecture in the absence of RingoA

**Figure 8:**
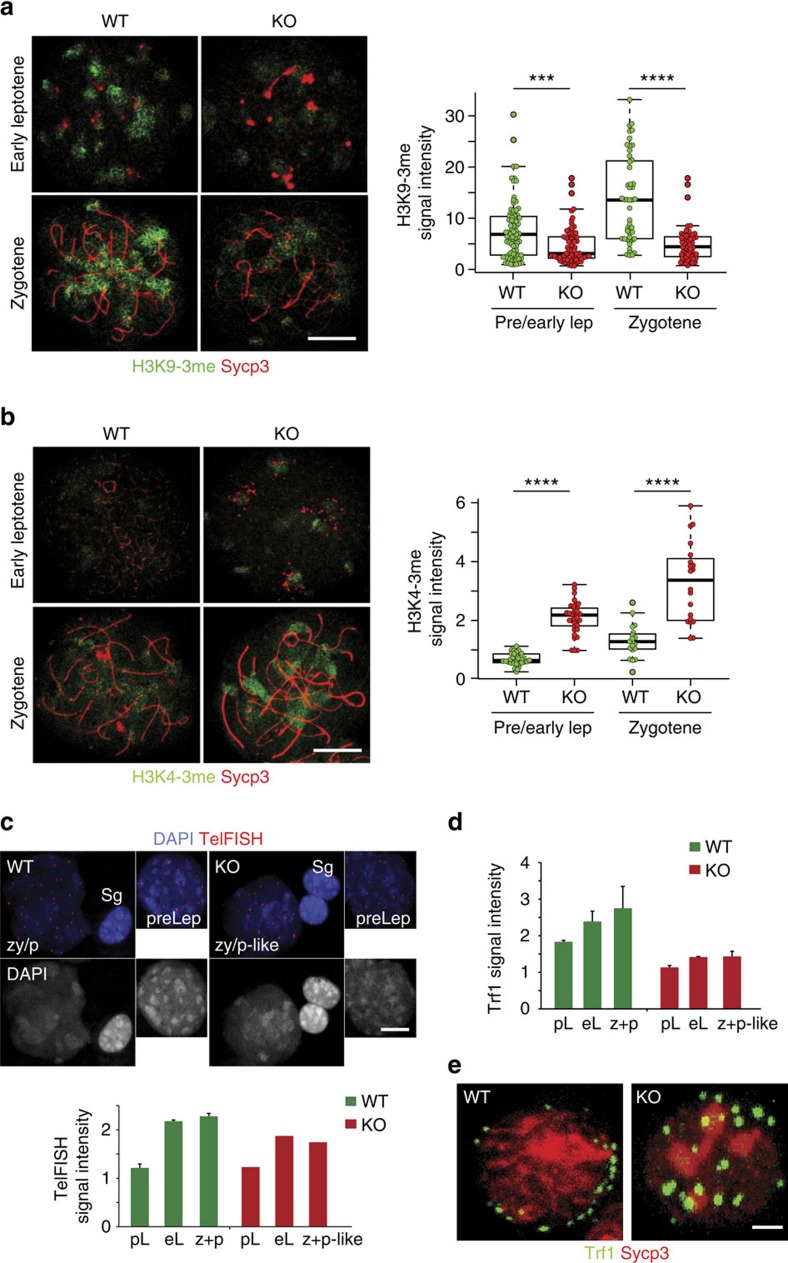
Meiotic telomere architecture is disrupted in the absence of RingoA. WT and KO spread spermatocytes were immunolabeled for Sycp3 (red) and either H3K9-3me or H3K4-3me (green in **a**,**b**). (**a**) KO spermatocytes showed weakened H3K9-3me staining concentrated in the chromocentres. The number of cells analysed for the quantification of the H3K9-3me signal intensity were 82 (WT) and 74 (KO) in pre/early Lep; 48 (WT) and 59 (KO) in zygotene. *P* value (pre/early Lep)=0.0002, *P* value (zygotene)=2.9e−9. (**b**) H3K4-3me immunolabeling in RingoA KO spermatocytes is enhanced at chromocentres. The number of cells analysed for the quantification of the H3K4-3me signal intensity were 34 (WT) and 35 (KO) in pre/early Lep; 19 (WT) and 20 (KO) in zygotene. Results show the average with s.d. of the measured intensities per nucleus. *P* value (pre/early Lep)<2.2e−16, *P* value (zygotene)=2.7e−07. (**c**) Spread spermatocytes at preleptotene (preLep) and zygotene/pachytene (zy/p and zy/p-like) from 16 dpp WT and KO littermate mice were labelled by TelFISH and DAPI. TelFISH signal intensities per nucleus were normalized to the mean TelFISH signal intensities measured in spermatogonial nuclei of the same sample (*n*=2 per genotype, 25 Sg, 15 pL, 10 eL and 20 z+p-like cells per genotype were measured). Average with s.d. is represented. (**d**) Same samples as in **c** were immunolabelled for Trf1, Sycp3 and DAPI. Trf1 signal intensity was measured and normalized to Trf1 signal intensity in spermatogonial nuclei (Sg), as in **c**. Average with s.d. is represented. eL, early leptotene; pL, preleptotene; z+p and z+p-like, zygotene/pachytene. Scale bar, 10 μm. (**e**) Squashed late leptotene/zygotene spermatocytes from WT and KO testes were immunostained for Sycp3 (red) and Trf1 (green). Bouquet formation is observed in 76% of WT cells but in none of the KO cells (*n*=2 per genotype, ≥30 cells per mouse). Scale bar, 10 μm (**a**–**c**) and 2 μm (**d**). Asterisks (**a**,**b**) denote statistical significance as determined by the unpaired two-way Wilcoxon rank-sum test (***P value<0.001, ****P value <0.0001).

The lack of telomere attachment in RingoA KO spermatocytes could be explained by a lack of Sun1 interactions at the inner nuclear membrane, but it is unlikely to account for the telomere fusions observed in pachytene-like spermatocytes since Sun1 KO mice do not show this phenotype^36^. Interestingly, Cdk2 KO spermatocytes show telomere fusions and changes in distribution of histone H3 trimethylated on Lys9 (H3K9-3me) (ref. 29), a histone modification involved in telomere maintenance^41^ as well as in homologous pairing during early prophase I^42^,^43^. We found H3K9-3me staining highly concentrated at chromocentric regions of pachytene WT spermatocytes, which was significantly reduced in RingoA KO spermatocytes from early leptotene (Fig. 8a). H3K9-3me chromatin is believed to be more compacted and therefore in a repressed state^44^. Furthermore, histone H3 trimethylated on Lys4 (H3K4-3me), which usually indicates active chromatin^45^, was substantially increased in RingoA KO spermatocytes (Fig. 8b).

Loss of telomere compaction and fusion can be connected to telomere shortening^46^ or in the meiosis context to the lack of proper elongation^41^,^33^. We assessed telomere length by measuring the intensity of telomeric FISH signals and Trf1 immunostaining signal in WT and RingoA KO spermatogonia and spermatocytes throughout prophase I. We found that the WT spermatocytes had progressively longer telomeres as prophase I progressed, compared with the spermatogonia (Fig. 8c,d). This trend was maintained in RingoA KO spermatocytes, but to a much lesser degree, and RingoA KO spermatocytes overall had shorter telomeres than the WT spermatocytes at the same stage (Fig. 8c,d).

We hypothesized that shorter and more ‘open' chromatin at the telomere could render them fragile, so that telomeres would get torn and damaged during the attachment and pulling of early leptotene to try to form the ‘bouquet' leading to the observed effects. Therefore, we assessed whether the RingoA KO spermatocytes retain the ability for telomere attachment and bouquet formation in late leptotene/zygotene using squashed 10 dpp spermatocytes. We were able to detect bouquets in 76% of WT spermatocytes but none in RingoA KO (Fig. 8e). These results indicate RingoA KO meiotic telomeres are essentially non-functional with reduced length, impaired chromatin compaction and largely unable to attach to the NE, as it has been reported in Cdk2 KO spermatocytes^37^.

## Discussion

Our results show that RingoA KO mice have a meiotic phenotype that is strikingly similar to that shown for Cdk2 KO mice^17^,^18^,^29^,^37^. In both cases, adult mice have no overt phenotype but both male and female are sterile. Moreover, RingoA or Cdk2 KO spermatocytes are arrested in a pachytene-like stage and show non-homologous pairing with partner switching and telomere attachment failure, DSB repair block and sex-body absence. Importantly, RingoA co-localizes at telomeres with Cdk2 and the telomeric localization of Cdk2 is impaired in RingoA KO spermatocytes. Taken together, our results indicate that RingoA is a likely key activator of Cdk2 in the prophase I of male and female meiosis, and that Cdk2-RingoA is critical for telomere maintenance and tethering to the NE as well as for accurate SC formation.

The idea that the meiotic Cdk2 function depends on RingoA is further supported by the observations that no cyclins have been detected at meiotic telomeres and that none of the cyclin KO mice reported so far have exactly the same phenotype as the male and female Cdk2 KO mice^19^,^20^,^22^,^23^,^47^. Recent work has shown that the combined deletion of cyclins E1 and E2 results in male infertility with impaired DSB repair, loss of telomere integrity and Cdk2 mislocalization but, in contrast to Cdk2 KO mice, females are fertile^21^. This suggests that cyclins E1 and E2 might function in the same pathway as RingoA during spermatogenesis, perhaps contributing to the telomeric location of the Cdk2-RingoA complex or as part of a more tightly regulated checkpoint control. As female meiosis is less stringent, Cdk2-RingoA activity might suffice, in the absence of cyclins E1 and E2, for some cells to complete prophase I.

The complex phenotype observed upon RingoA depletion suggests that RingoA either can independently regulate several processes or maybe just controls a key event whose deregulation triggers the rest. By comparing the phenotypes of mouse mutants that are affected in prophase I of meiosis, we noted that the RingoA KO phenotype is very similar to that of the Sun1 KO mice. Absence of Sun1 produces a meiotic phenotype with aberrant telomere attachment to the NE and SC formation, including non-homologous pairing and accumulation of unrepaired DSBs in pachytene spermatocytes^36^. It has been recently shown that some telomeres in Sun1 deficient spermatocytes, which are located close to the NE, are still connected via Sun2 and can move to form the bouquet, strongly suggesting that full telomere attachment is required to support progression through prophase I^48^. We have found that RingoA deficiency abrogates the telomeric localization of Sun1, but not that of Terb1, which connects Sun1 to Trf1 (ref. 34). Thus, RingoA-dependent Sun1 recruitment to the telomere could be a crucial function whose disruption might entail all the downstream phenotypes that could be observed in RingoA KO mice. How RingoA controls the localization of Sun1 is currently unknown. The ability of endogenous Cdk2 to bind to Sun1 (ref. 49) and of recombinant Cdk2-RingoA to phosphorylate the N-terminus of Sun1, suggests a phosphorylation-dependent mechanism. It should be noted that although loss of Sun1 might explain several features of the RingoA-deficient spermatocytes, it is unlikely to explain the telomere fusion phenotype.

The reduction in heterochromatin markers and FISH signals on telomeres suggests reduced chromatin compaction and diminished elongation of telomeres in RingoA-depleted spermatocytes. Telomere elongation can be achieved by two mechanisms, *de novo* synthesis by telomerase and elongation by recombination (ALT). The ALT mechanism has been studied in telomerase negative tumour cells and was shown to involve DSB repair proteins^50^ and Cdk2 (ref. 41). It is interesting that KO mice for the topoisomerase-like enzyme Spo11 or the repair proteins Msh5 and Dmc1 (ref. 43) all show meiotic chromosome fusions, although detailed studies are missing. A study in mouse ES cells has proposed that Spo11 and Dmc1 might work in telomere maintenance outside of the meiosis context^51^ and the ‘meiotic' pathway could have adapted to maintain telomeres in tumours^52^. Homologous recombination in general requires Cdk1 and Cdk2 activity^53^,^54^. In somatic cells, this function is carried out by cyclin A2 but mitotic cyclins and Cdk1 should be maintained at low levels during prophase I to avoid premature entry into pro-metaphase I. Thus, RingoA could substitute for cyclin A in prophase I and perhaps do the same function in somatic cells whenever cyclin A expression is low, for example when DNA damaged cells are arrested in G2-phase. It would be interesting to test whether RingoA has a function in telomere maintenance in telomerase negative tumours or in homologous recombination.

In summary, our work identifies the atypical CDK activator RingoA as a physiological regulator of Cdk2 in prophase I of meiosis and supports a role for Cdk2-RingoA in the establishment of functional meiotic telomeres, NE tethering and homologous recombination. Although Cdk2 can be potentially activated by many cyclins and several of them are expressed in meiotic prophase I, RingoA seems to be the only Cdk2 activator that is expressed at the right time (leptotene to pachytene) in the right place (telomeres and sex-body), and that shows the same loss-of-function phenotype as Cdk2.

## Methods

### Generation and analysis of RingoA KO mice

To generate mice deficient in RingoA, we constructed a targeting vector with two loxP sites flanking the exon 3 of the *Spyda* gene encoding RingoA (Supplementary Fig. 1). Splicing from exons 2 to 4 is predicted to cause a frameshift that results in a premature STOP codon. The targeting vector was generated by Gene Bridges GmbH (Heidelberg, Germany) using a C57BL/6 bacterial artificial chromosome and was verified by sequencing. ES cells (129/SVJ) were electroporated with the linearized targeting vector and correct chromosomal insertion was verified by Southern blot analysis of NcoI-digested genomic DNA using external probes. Injection of a positive clone into blastocysts generated chimeras, which transmitted the recombinant locus. *Spdya*^lox/+^ mice were crossed with Sox2-Cre mice to remove the loxP-flanked exon 3 and generate *Spdya*^*+/−*^ mice, which were inter-crossed to produce RingoA KO animals. Mice were maintained in C57BL/6 background. Genotyping was performed by PCR using tail genomic DNA and primers: p1 (5′-TGGGCCATTAGCATTTTGTGAGCT-3′) and p2 (5′-TGCTTTGGGGCCAGTGAGATGA-3′) to detect the WT allele (298 bp); p1 and p3 (5′-GGCTGCTAAAGCGCATGCTCCA-3′) to detect the floxed allele (197 bp); p4 (5′-GCCGCATAACTTCGTATAAT-3′) and p5 (5′-CCACCACTCTGGGATAGATA-3′) to detect the KO allele (296 bp). The Cre transgene was detected using the primers CreF (5′-CCCACCGTCAGTACGTGAGAT-3′) and CreR (5′-GTGGCAGATGGCGCGGCAACAC-3′) (450 bp). Fertility was assessed by keeping KO mice with WT C57BL/6 mice of the opposite sex over 3–6 months. The C57BL/6 partners were subsequently mated with WT animals and gave birth to offspring. Mice were housed in a temperature-controlled facility using individually ventilated cages, standard diet and a 12 h light/dark cycle, according to national and European Union regulations. Mouse protocols were approved by the Ethics Committee for Animal Experimentation of the Barcelona Science Park (CEEA-PCB). We made every effort to minimize and refine our experiments to avoid animal suffering. Blinded experiments were not possible since the phenotype was very obvious between WT and RingoA KO samples for all of the experimental procedures used.

### Histology and TUNEL staining

For histological examination, whole mouse testes were fixed in Bouin's solution (Electron Microscopy Sciences) and ovaries in 10% paraformaldehyde (PFA, neutral buffered, Sigma), at 4 °C overnight, washed in PBS, and then were paraffin embedded and stained with hematoxylin and eosin (H&E). For apoptosis detection, testis were fixed in 10% PFA (neutral buffered, Sigma) and sections (9 μm) were stained using the TUNEL *in situ* Cell Death Detection Kit (Roche, 11684795910) following the provided protocol.

### Meiotic spreads and squashes

Testes from 16 to 22 dpp WT and RingoA KO littermate mice were used for spermatocyte spreads and seminiferous tubules squashes, unless indicated otherwise. Tissue was macerated using a razor blade and the pieces were collected in 1 ml of Dulbecco's Modified Media (DMEM) supplemented with protease inhibitor cocktail (1 tablet per 50 ml of DMEM, Roche, 11873580001). After thorough resuspension using a pipette, 5 ml of DMEM were added and the mixture was left on ice until the big pieces settle at the bottom of the tube. The supernatant (1 ml) was put into an Eppendorf tube and spun down for 5 min at 5,000*g* at room temperature. The supernatant was removed and the pellet was resuspended in 40 μl of 0.1 M sucrose solution supplemented with protease inhibitors. We dropped 20 μl of this solution onto slides previously layered with 65 μl of 1% PFA, 0.1% Triton X-100 with protease inhibitors. The slides were allowed to dry and then were rinsed with Photoflo solution (1:250) in a Coplin jar. Stainings were usually performed right away or the slides were stored at −80 °C until further use.

Seminiferous tubules squashes were essentially done as in ref. 55 and immediately immunolabelled. In short, freshly extracted 10 dpp testes were minced into small pieces with tweezers in 2% PFA, 0.05% Triton X-100 and fixed for 10 min. A small piece was then put on each slide previously rinsed with ethanol:chloroform (1:1), covered with a coverslip and gently pressed to distribute the material. The slides were immersed into liquid nitrogen. Then the coverslip was removed, washed in PBS and used immediately.

### Immunostaining and telomere FISH

The spermatocyte spreads and seminiferous tubules squashes were blocked for 30 min with 1% BSA and 0.05% Tween in PBS (PBS-T) and then were incubated overnight at 4 °C with the following primary antibodies: Sycp3 (Abcam, ab15093 and ab97672, 1:800; and Santa Cruz, sc-20845, 1:20); Sycp1 (Abcam, ab15087, 1:800) Dmc1 (Santa Cruz, sc-22768, 1:50); Trf1 (Alpha Diagnostics, TRF12-S, 1:50); Sun1 (Abcam, ab103021, 1:50); H1t (gift from Dr Mary Ann Handel, The Jackson Laboratory, Bar Harbor, Maine, USA, 1:200) H3K9-3me (Milipore, 05-1250, 1:2000); γH2AX (Milipore, 05-636, 1:300); Mlh1 (Becton Dickinson, 551091, 1:100); Rad51 (Santa Cruz, sc-8349, 1:100) Terb-1 (affinity-purified rabbit antiserum, 1:100)^34^ and Cdk2 (Abcam, ab7954, 1:50). RingoA was detected using the R55A mouse monoclonal antibody^12^, which recognizes the sequence NDHQSNK corresponding to amino acids 283-289 (hybridoma supernatant, 1:1). The slides were then washed in blocking buffer and incubated for 1 h at 37 °C with appropriate Alexa Fluor-labelled secondary antibodies (Invitrogen, A21442, A31571, A21441, A21468, A21203, A11017, 1:300). Finally, the slides were washed and mounted in ProLong Gold antifade reagent (Molecular Probes).

For telomere FISH staining, Telomere PNA FISH kit/cy3 (Dako, K5326) was used on 16 dpp testis spreads following the protocol described by the manufacturer.

### Microscopy

Confocal images were taken on the Leica TCS SPE microscope (Leica, Mannheim, Germany) using an HCX PL APO lambda blue × 63, NA 1.3, oil UV objective, 405-, 488-, 543- and 635-nm laser excitation at a pixel resolution of 71 nm. Images were processed using ImageJ and Adobe Photoshop CS6.

### Transmission electron microscopy

Testes from 2-month-old mice were prepared as described in ref. 56. In short, the testes were collected and disintegrated with a scalpel to 3 × 3 mm pieces and fixed for 1 h at 4 °C in 2.5% glutaraldehyde solution in cacodylate buffer (50 mM cacodylate, 50 mM KCl, 2.5 mM MgCl2, pH 7.2). After five washes in cacodylate buffer, they were further fixed for 2 h at 4 °C in 2% osmium tetraoxide, washed three times in water, and incubated in 0.5% uranyl acetate overnight. Finally, the samples were dehydrated in ethanol and embedded in propylene oxide/Epon (1:1) at room temperature. Ultrathin sections were mounted on the copper grids, incubated with 2% uranyl-acetate at room temperature for 20 min, followed by counterstain in Reynold's lead citrate for 10 min. The images were acquired on TEM Jeol 1010 operated at 80 kV.

### Statistical analysis

Graphics with error bars represent average±s.d. The statistical significance was determined by unpaired two-way Wilcoxon rank-sum test. Scatterplots in Figs 1e, 3d and 6a,b show individual values with the average and s.d. indicated (except in Fig. 1e). Asterisks denote statistical significance: **P value ≤0.01, ***P value ≤0.001 and ****P value≤0.0001.

### Immunoprecipitation and immunoblotting

Mouse testes were lysed in cold IP buffer containing 20 mM Tris-HCl (pH 7.5), 150 mM NaCl, 1% NP-40, 5% glycerol, 5 mM 6-(Dimethylamino) purine, 10 mM EDTA, 5 mM NaF, 1 mM Na_3_VO4, 1 mM DTT, 1 μM microcystin, 25 μM MG132, 400 μM PMSF, 10 μg ml^−1^ each of aprotinin, leupeptin and pepstatin. Lysates were centrifuged at maximal speed (Eppendorf) for 10 min at 4 °C and the supernatants were used for immunoprecipitation or immunoblotting.

Immunoprecipitations were performed using bead-coupled commercial antibodies against Cdk2 (Santa Cruz, sc-163 AC) and either control rabbit IgGs or RingoA rabbit antiserum (#1870, raised against the peptide AVRNYNRDEVHLPRGP corresponding to amino acid 205-220) that were coupled to protein G beads (Pierce, 20398) with DMP (Sigma, D8388). Bead-coupled antibodies were incubated for 1 h at 4 °C with lysates of testis (3.3 mg) and then were washed twice with IP buffer and twice with IP buffer without NP-40 and glycerol.

For immunoblotting, samples were separated by SDS–polyacrylamide gel electrophoresis (SDS–PAGE), transferred to nitrocellulose and incubated for 1 h at room temperature with antibodies against RingoA (R55A hybridoma supernatant, 1:10) or Cdk2 (Santa Cruz, sc-163-G, 1:1,000). Membranes were washed three times with PBS-T for 10 min. Immunoblots were developed using antibodies labelled with Alexa Fluor 680 (Molecular Probes) or Li-cor IRDye 800 (Rockland) (1:5,000) and the Odyssey Infrared Imaging System (Li-Cor). Original scans of the immunoblots indicating the cropped area are provided in Supplementary Fig. 7.

### Recombinant protein production

Human Sun1 amino acids 1-217, either WT or the S48A mutant, and amino acids 456–916 were fused to MBP^40^. Human Cdk2 (ref. 6) and human RingoA2 (ref. 10) were fused to GST. Recombinant proteins were purified by standard methods^6^. In brief, a single colony transformed with the appropriate construct was grown in 5 ml LB medium with 100 μg ml^−1^ ampicillin overnight at 37 °C. The culture was then diluted 1:100 into 500 ml fresh medium and incubated for 4 h at 37 °C followed by the addition of 1 mM IPTG for 3 h at 37 °C. Cells were collected in 25 ml of ice-cold PBS containing 0.2% Triton X-100, 1 mg ml^−1^ Lysozyme, 100 μM PMSF, 10 μg ml^−1^ each of aprotinin, leupeptin and pepstatin and were sonicated 6 × 15 s. Lysates were centrifuged at maximal speed for 10 min and supernatants were transferred to another tube containing glutathione-agarose beads (GE Healthcare, 17–5132) or dextrin sepharose beads (GE Healthcare, 28-9355-97), and were rotated for 1 h at 4 °C. Beads were collected by centrifugation and washed four times in 10 ml of ice-cold PBS-T. GST proteins were eluted in 20 mM glutathione and 50 mM Tris-HCl pH 8.0, and MBP proteins in 10 mM Maltose and 10 mM Tris-HCl at pH 7.5.

### Kinase assays

For *in vitro* kinase assays, purified MBP-Sun1 proteins (1 μg) were incubated in a final volume of 30 μl of kinase buffer (50 mM Tris-HCl at pH 7.5, 10 mM MgCl_2_, 1 mM DTT, 3 μM cold ATP, 1 mM NaF, 1 mM Na_3_VO_4_, 200 μΜ PMSF, 10 μg ml^−1^ each of aprotinin, leupeptin and pepstatin) containing 5 μCi of [γ-^32^P]ATP with GST-Cdk2 (0.5 μg) and GST-RingoA (0.5 μg) for 90 min at 37 °C. The reactions were stopped by adding sample buffer and boiling, and were analysed by SDS–PAGE and autoradiography.

To assay the kinase activity of the endogenous Cdk2, testes from 20 dpp WT or RingoA KO mice were homogenized with the Precellys 24 tissue homogenizer (Bertin technologies) in IP buffer (50 mM Tris-HCl at pH 7.5, 50 mM NaCl and 0.5% Nonidet P-40) supplemented with protease inhibitor cocktail (Millipore 539134). Homogenized lysates were clarified by centrifugation and the supernatant (1 mg) was incubated with 15 μg of a rabbit anti-Cdk2 antibody-conjugated agarose beads (Santa Cruz Biotechnology, sc-163 AC) for 2 h at 4 °C. Immunoprecipitates were washed three times with 1 ml of IP buffer and three times with kinase buffer (50 mM Hepes pH 7.5, 50 mM urea, 10 mM MgCl2, 5 mM MnCl2 and 1 mM DTT). The beads were suspended in 30 μl of kinase buffer containing 1 μg of Rb protein (amino acids 773-928, Millipore, 12-439) and 10 μCi of [γ-^32^P]ATP (Perkin Elmer). After incubation for 30 min at 30 °C with mixing, reactions were stopped with sample buffer and analysed by SDS–PAGE and autoradiography.

### Real-time quantitative PCR

Dissected testes were collected and snap frozen in liquid nitrogen. For total RNA preparation, the testes were disrupted in lysis buffer with the mechanical tissue disruptor (Precellys 24, Bertin Technologies) using the Ambion PureLink RNA mini kit. The reverse transcriptase reaction was performed with 4 μg of RNA. Amplification of the cDNA was done on a thermocycler (C1000 Thermal Cycler, Bio-Rad). *Spdya* expression was detected using three sets of primers to amplify a fragment corresponding to amino acids 85–195 (including the deleted exon 3): p1 (5′-TTCTTGTGGATGGACTGCTG-3′) and p2 (5′-TTGCCAGATGTAATGGGTTG-3′), the 5′UTR: p3 (5′-GCAACCGCCTGAGGTAGAT-3′) and p4 (5′-TGATTATGCCGCATTTTAGC-3′), and a C-terminal fragment downstream of exon 3 (corresponding to amino acids 147–187): p5 (5′-GGGCTTTAGGGAAAAACTGG-3′) and p6 (5′-AATGGCCATGACCTCTTCAC-3′). As control, we used primers to amplify the housekeeping genes Gapdh: p7 (5′-CTTCACCACCATGGAGGAGGC-3′) and p8 (5′- GGCATGGACTGTGGTCATGAG-3′), and Hprt: p9 (5′- TCAGTCAACGGGGGACATAAA -3′) and p10 (5′- GGGGCTGTACTGCTTAACCAG-3′). Data were plotted using Bio-Rad CFX Manager. The amplification products were analysed by 2−ΔΔCT method.

## Additional information

**How to cite this article:** Mikolcevic, P. *et al.* Essential role of the Cdk2 activator RingoA in meiotic telomere tethering to the nuclear envelope. *Nat. Commun.* 7:11084 doi: 10.1038/ncomms11084 (2016).

## Supplementary Material

Supplementary Information Supplementary Figures 1-7 and Supplementary Table 1

Supplementary Movie 1 Telomeres of WT spermatocytes attach to the NE. 3D reconstruction of squashed pachytene spermatocyte immunolabeled for Sycp3 (red) and Trf1 (green). Trf1 is visible at the edges of the cell nuclei, i.e., the ends of Sycp3 threads.

Supplementary Movie 2 Telomeres of KO spermatocytes fail to attach to the NE. 3D reconstruction of squashed Ringo A KO pachytene-like spermatocyte immunolabeled for Sycp3 (red) and Trf1 (green). Trf1 is visible both at the edges of the cell nuclei and inside the nucleus.
