# Supplementary material for: Essential role of the Cdk2 activator RingoA in meiotic telomere tethering to the nuclear envelope
Source: Nat Commun. 2016 Mar 30;7:11084. doi: 10.1038/ncomms11084 (PMC4820962; doi:10.1038/ncomms11084)
Supplement: Supplementary Information — Supplementary Figures 1-7 and Supplementary Table 1 [file ncomms11084-s1.pdf]

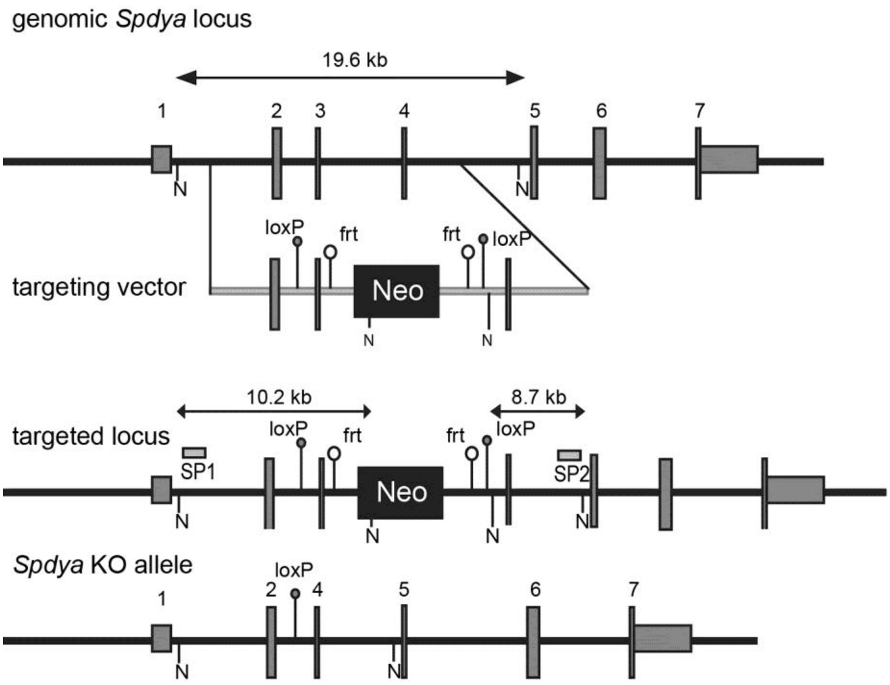


**Supplementary Figure 1. Generation of RingoA KO mice.** The targeting vector contained a 5.7 Kb 5’ homology arm, a *loxP*-flanked fragment containing exon 3 of the *Spdya* gene encoding RingoA followed by an *frt*-flanked neomycin resistance (*neo*) cassette and a 6.6 Kb 3’ homology arm. N: NcoI; SP1: 5’ probe; SP2: 3’ probe.

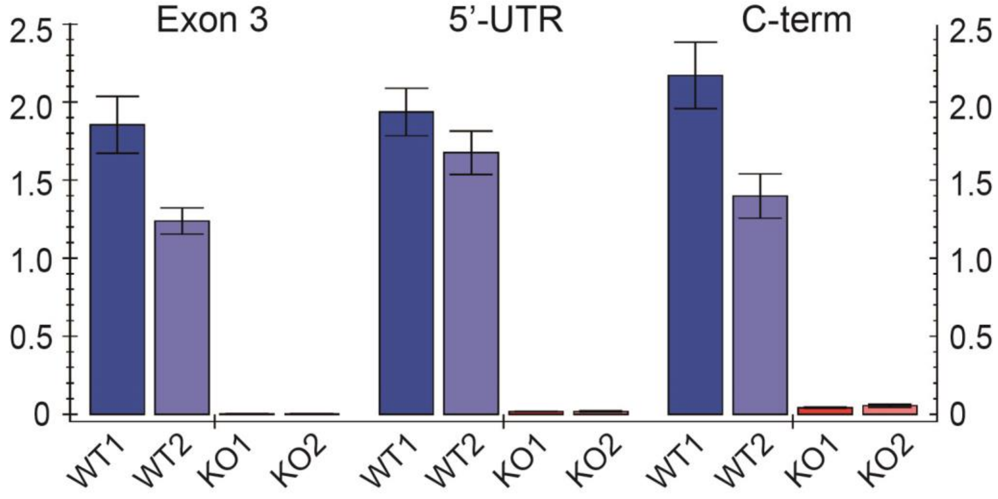


**Supplementary Figure 2. Analysis of RingoA mRNA expression.** Quantitative real-time PCR of *Spdya* mRNA using cDNA prepared from 2 months-old testes of WT and RingoA KO animals and three sets of primers to amplify a fragment corresponding to amino acids 85-195 (including the deleted exon 3), the 5'UTR, and a C-terminal fragment downstream of exon 3 (corresponding to amino acids 147-187). The level of *Spdya* expression is represented as normalized to the housekeeping genes *Hprt* and *Gapdh*. Error bars represent s.d.

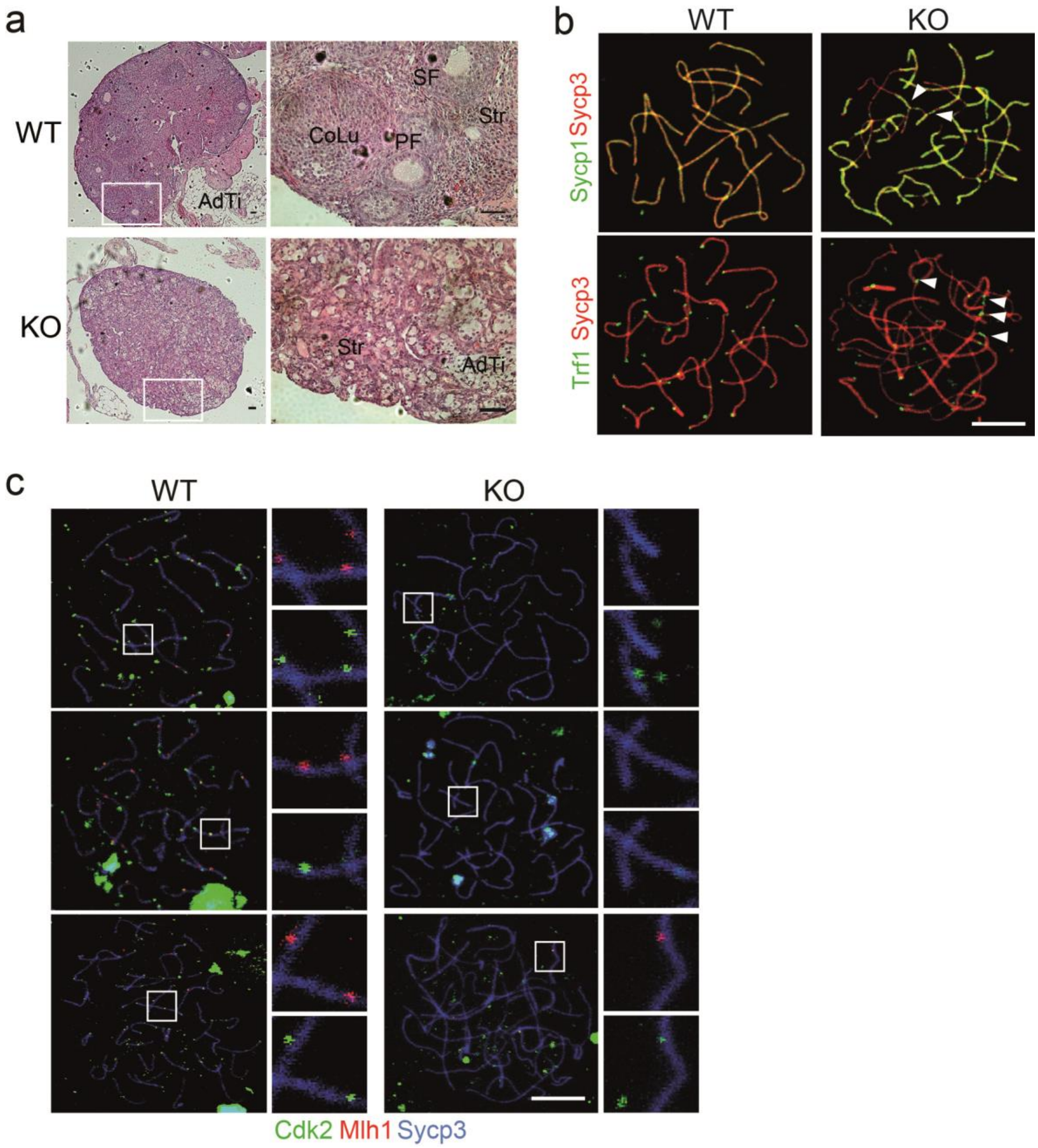


**Supplementary Figure 3. RingoA KO ovaries are atrophic and oocytes lack proper synapsis with partner switching and telomere fusions. (a)** Histological sections of ovaries from three month-old females were stained with H/E. WT ovaries show normal follicle development while KO are totally atrophic without follicles. AdTi, adipose tissue; Str, stroma; PF, primary follicle; SF, secondary follicle; CoLu, corpus luteum. (**b**) Pachytene/pachytene-like spread oocytes (1 dpp) from WT and KO ovaries were stained for Sycp3 (red) and Sycp1 or Trf1 (green). Arrowheads indicate non-homologous pairing and partner switching in the first row, and telomere fusions in the second row. (**c**) Pachytene/pachytene-like spread oocytes (1dpp) from WT and KO ovaries were stained for Sycp3 (blue), Cdk2 (green) and Mlh1 (red). Size bars = 100 µm (**a**), 10 µm (**b** and **c**).

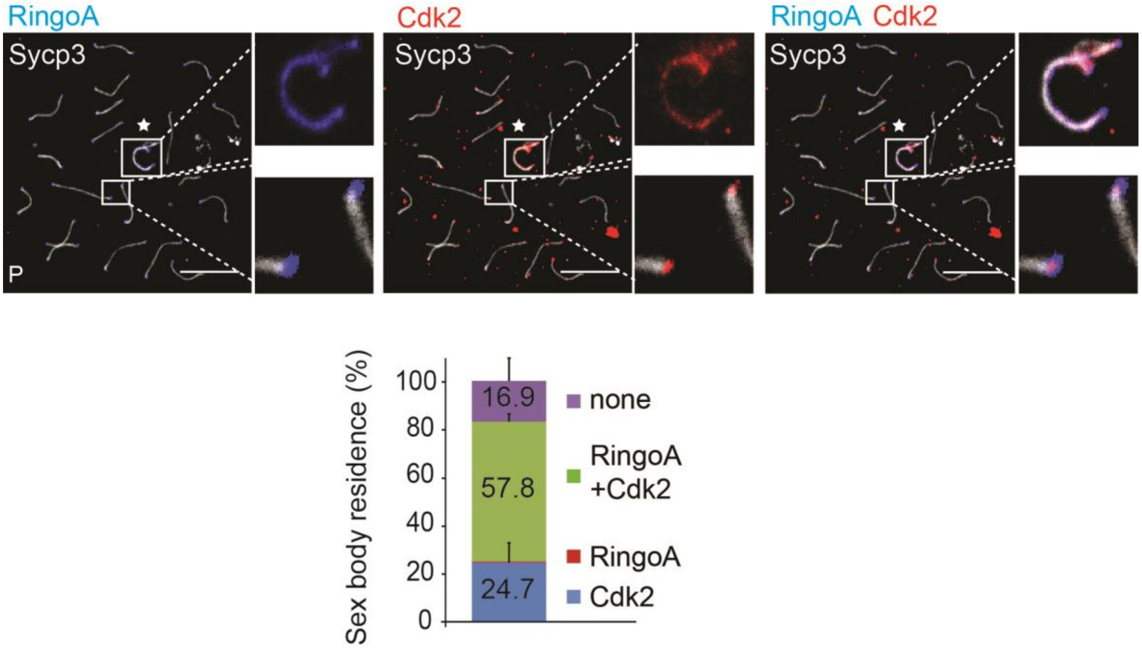


**Supplementary Figure 4. Cdk2 and RingoA co-localize at telomeric regions as well as the axial elements of sex chromosomes in pachytene spermatocytes**. WT pachytene spread spermatocytes were immunolabeled for Sycp3 (white), RingoA (blue) and Cdk2 (red). Stars indicate the sex bivalent. Quantification of RingoA and Cdk2 localization at the axial elements of sex chromosomes in WT pachytene spermatocytes (n=166, 3 mice). Error bars represent s.d.

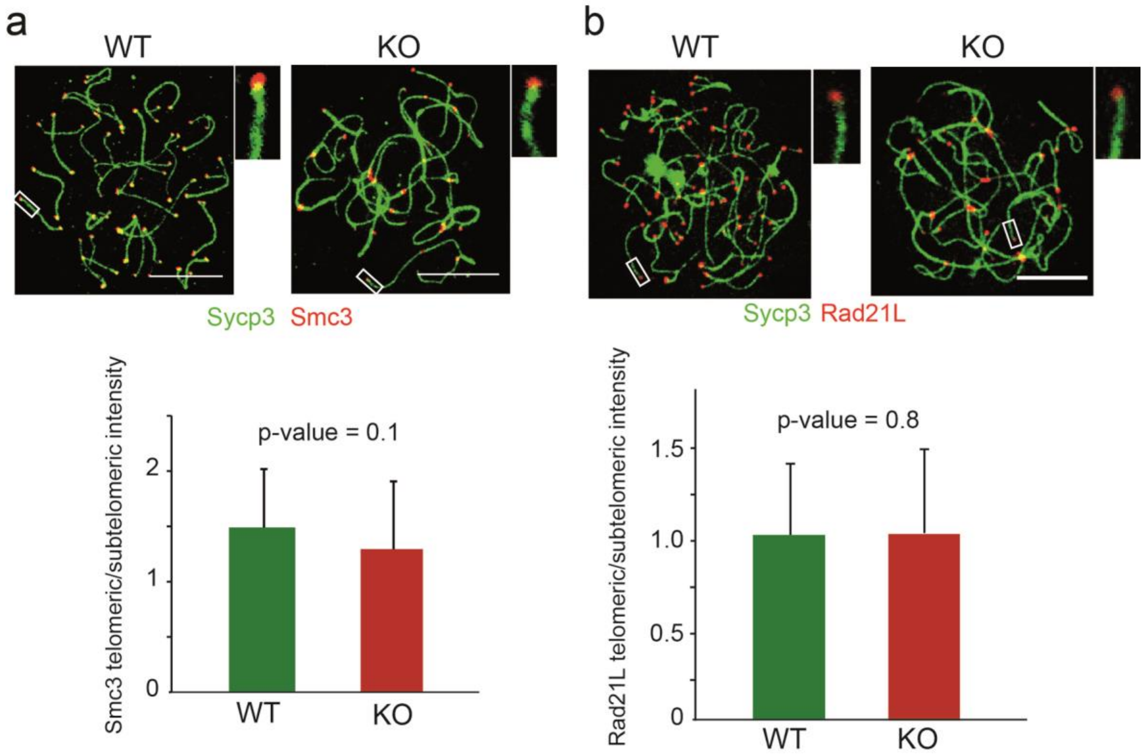


**Supplementary Figure 5. RingoA deletion does not affect enrichment of cohesins at the telomere.** WT and KO spread spermatocytes were immunelabelled for Trf1 (determining the telomere position) and either Smc3 or Rad21L. (**a**) Quantification of the mean intensity of Smc3 signal at the telomere normalized to Smc3 signal at the adjacent arm. Three telomeres per cell were quantified (n= 10 cells). (**b**) Quantification of the mean intensity of Rad21L signal at the telomere normalized to Rad21L signal at the adjacent arm. Three telomeres per cell were quantified; n= 20 (WT) and 15 (KO) cells. Error bars represent s.d.

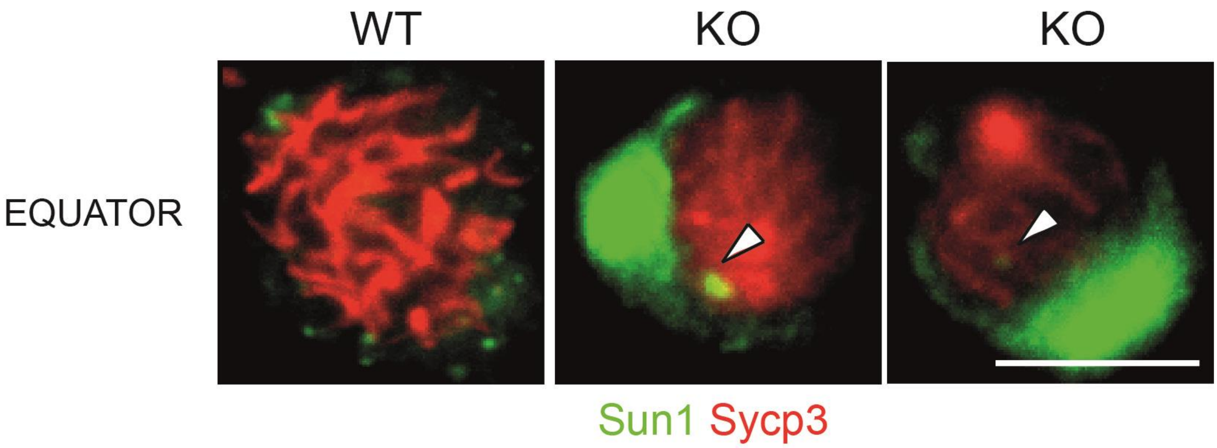


**Supplementary Figure 6. Sun1 staining at the spermatocyte equator.** WT and KO squashed spermatocytes were immunolabeled for Sun1 (green) and Sycp3 (red). Arrowheads indicate internal Sun1 signals. Scale bar = 5 µm.

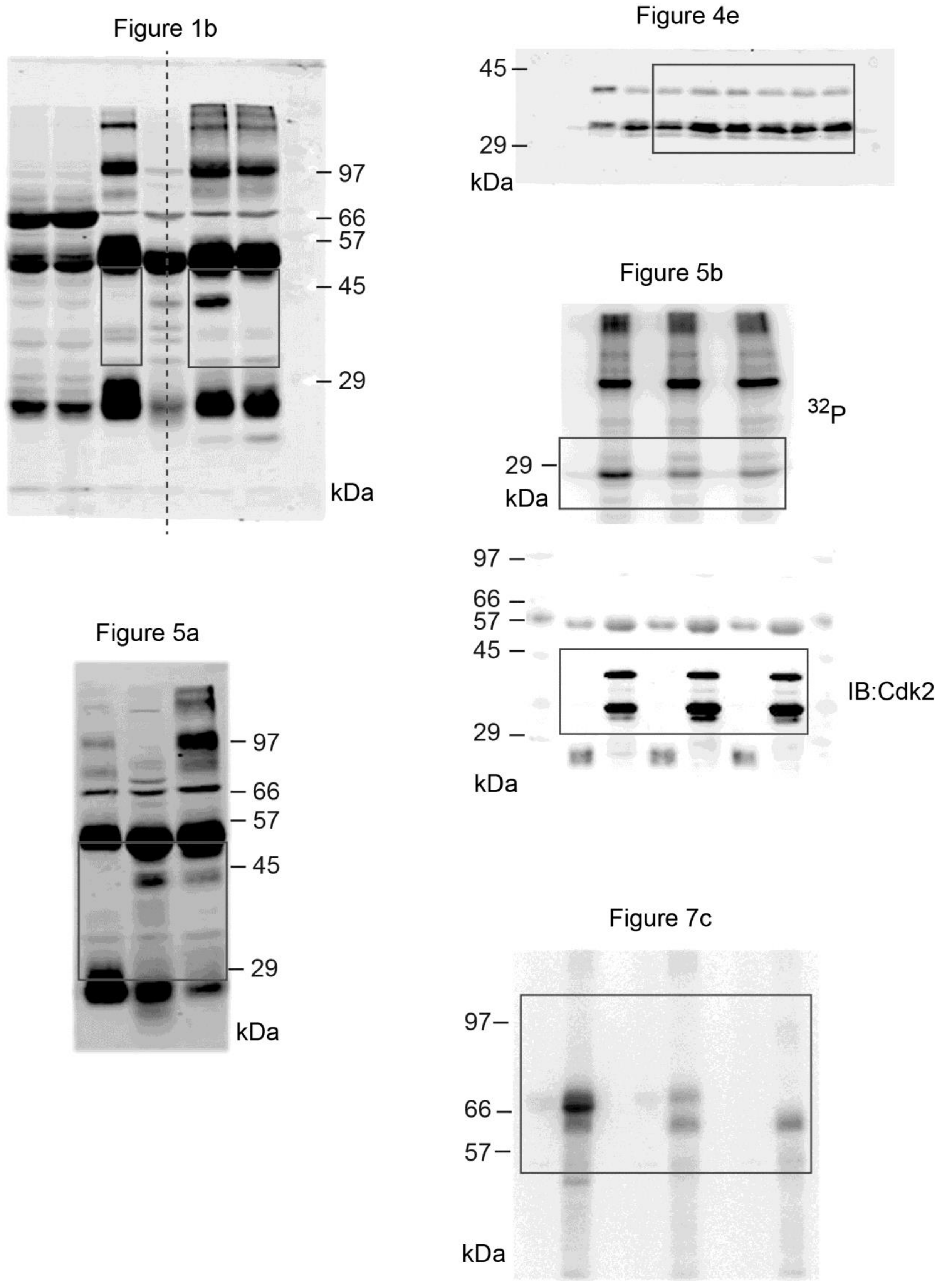


**Supplementary Figure 7.** Uncropped immunoblots and autoradiographies of the indicated figures.

**Supplementary Table 1**. Observed and expected frequencies of genotypes in mice born from breeding heterozygous $RingoA^{+/-}$ mice.

| | Wild type | Heterozygous | Knockout |
|---|---|---|---|
| **Observed (no)** | (63) 24% | (130) 51% | (62) 25% |
| **Expected** | 25% | 50% | 25% |
| $\chi^2$ | 0.9 | | |

A total of 255 mice were genotyped.
